# Antibody Phage Display Libraries: Contributions to Oncology

**DOI:** 10.3390/ijms13055420

**Published:** 2012-05-04

**Authors:** Carmela Dantas-Barbosa, Marcelo de Macedo Brigido, Andrea Queiroz Maranhao

**Affiliations:** 1 Centre de Recherche en Cancérologie de Lyon, UMR INSERM 1052-CNRS 5286, Centre Léon Bérard, Cheney D, 28 Rue Laënnec, 69373 Lyon Cedex 08, France; 2 Laboratório de Biologia Molecular, CEL/IB, Universidade de Brasília, Campus Darcy Ribeiro, 70910-900, Brasília-DF, Brazil; E-Mails: brigido@unb.br (M.d.M.B.); andreaqm@unb.br (A.Q.M.)

**Keywords:** phage display, tumor markers, clinical antibodies

## Abstract

Since the advent of phage display technology, dating back to 1985, antibody libraries displayed on filamentous phage surfaces have been used to identify specific binders for many different purposes, including the recognition of tumors. Phage display represents a high-throughput technique for screening billions of random fusion antibodies against virtually any target on the surface or inside cancer cells, or even soluble markers found in patient serum. Many phage display derived binders targeting important tumor markers have been identified. Selection directed to tumoral cells’ surfaces lead to the identification of unknown tumoral markers. Also the improvement of methods that require smaller amounts of cells has opened the possibility to use this approach on patient samples. Robust techniques combining an antibody library displayed on the phage surface and protein microarray allowed the identification of auto antibodies recognized by patient sera. Many Ab molecules directly or indirectly targeting angiogenesis have been identified, and one of them, ramucirumab, has been tested in 27 phase I–III clinical trials in a broad array of cancers. Examples of such antibodies will be discussed here with emphasis on those used as probes for molecular imaging and other clinical trials.

## 1. Monoclonal Antibodies, Useful Diagnostic and Therapeutic Molecules

Antibodies have been used for antigen detection and therapeutics, and their specificity combined with low toxicity make them a promising pharmaceutical commodity [1]. Actually, they comprise the second-largest category of biological medicines in clinical development, after vaccines [2]. Successful examples include rituximab, approved by the FDA since 1997, an anti-CD20 chimeric antibody that become an integral component of many treatment strategies for non-Hodgkin’s lymphoma [2], and OKT3, an anti-CD3 that is used to reduce graft rejection [3]. Therapeutic use of antibodies is limited by methodological constraints in raising them. They are commonly obtained by immunization of experimental animals, usually mouse, with target antigens. The screening leads to a specific antibody-producing hybridoma [4]. Although well established, this technology is laborious, and it is biased by the experimental animal immune system, which limits the ability to reach a high-affinity antibody against conserved mammal proteins. Additionally, the heterologous character of those proteins turn them often immunogenic to humans eliciting HAMA response (Human Anti-Mouse Antibodies), which restrict their therapeutic use [5]. Human antibodies are of particular interest due to a lower immunogenicity response [1]. Antibody humanization bypasses this bottleneck, minimizing the HAMA response by replacing murine sequences with human framework homologous sequences [6]. The challenge is to avoid immunogenicity and maintain the specificity. Humanized antibodies are a reality in many diseases treatments nowadays, including cancer therapy and diagnosis. Some successful examples are Herceptin, used for breast cancer therapy; Avastin, used in clinics to treat colorectal cancer; Lucentis, an anti-VEGF antibody, among others. Antibodies proprieties are incontestable; nevertheless, some applications can be limited by their molecular size. They present poor penetration into tissues (e.g., solid tumors) and poor or absent binding to functionally important regions on the surface of some molecules by sterical constrains. Antibody fragments such as Fab (fragment antigen-binding), scFv (single-chain variable fragment) are alternatives to decrease an antibody’s size and increase its penetration into tissues. Therefore, several efforts have been employed to develop novel smaller sized scaffolds. Scaffolds derived from single antibody domains can be about 10-fold smaller than full size antibodies [7,8]. Single-domain antibodies (sdAbs or dAbs), molecules containing either heavy chain or light chain variable regions of human antibodies, and nanobodies derived from the variable regions of HCAbs (heavy chain-only antibodies) of camelids [9], are the smallest antigen binding fragments tested.

A suitable approach to obtain human antibodies and their fragments is the construction and selection of human antibody combinatorial libraries displayed on filamentous phage surfaces [10]. These libraries can be synthetic or obtained from human patient repertoires and the selection of binders is performed targeting a previously defined ligand, resulting in a completely human antibody fragment, theoretically less immunogenic than murine or humanized ones. Antibody phage display indeed has generated numerous human recombinant antibodies for research, diagnostics and therapy. In 2003, the FDA approved the first phage display antibody, Adalimumab (Humira), to treat rheumatoid arthritis. Currently it is used also to treat psoriasis, arthritis, ankylosing spondylitis and Crohn’s disease, and clinical trials to expand its utilization to other diseases are under way [11]. Other examples, in Phase I or II clinical trials include human antibodies against HIV (a combination of three human antibodies: C2F5, C2G12 and C4E10; phases I and II, NCT00219986), GM-CSF (MT-203, Phase I, NCT01317797) and CD20 (ofatumumab, phase II for treat patients that are rituximab refractory, NCT00394836), tested for treatment of different diseases [12].

The purpose of this review is to depict the utilization of phage display libraries in selecting specific tools to be used in cancer research. Examples of Fab, scFv, sdAb or others antibody fragments derived from naïve or immunized libraries will be addressed. Binders used on research, in preclinical analysis and as molecular imaging probes will be illustrated. Finally, we will explore clinical trials using antibodies obtained from phage display libraries.

## 2. Antibody Phage Display Libraries

Phage display was established by George P. Smith in 1985 and describes the presentation of exogenous peptides on the coat of phage particles. It is based on the observation that bacteriophages from the *Inoviridae* family (M3, fd, fl) are properly packaged and retain infectivity even in the presence of foreign peptides fused to their capsid proteins [13]. A phage display library is obtained by genetic manipulation. Billions of peptides, protein variants, antibody fragment coding genes are cloned into a vector fused to the 5′ of one of the filamentous bacteriophage coat protein (pIII or pVIII) genes. Those phagemid libraries are used to transform bacteria which will be also infected with the helper phage. The helper phage infection allows the assembling of viral particles displaying the fusion proteins on their surface [14]. Since the coat protein provides only an anchor for the displayed peptide it should not interfere with its structure and allows affinity purification of the peptide and its corresponding gene. This rational also applies for antibody fragments presentation on the surface of filamentous phage [10,15,16].

Different strategies can be employed to obtain an antibody combinatorial phage display library. There are some synthetic or semi-synthetic libraries, constructed based on human variable light (VL) and variable heavy (VH) repertoires. The most used ones are Griffin-1 library (H Griffin, MRC, Cambridge, UK), composed by the majority of human VH and VL gene segments used *in vivo*, with additional CDR3 diversity generated using synthetic oligonucleotides (semi-synthetic) and Tomlinson I Library [17] that consists of a single human framework with diversity incorporated at 18 amino acid positions among VH and VL domains, mainly in the antigen binding site (synthetic). In both libraries the antibodies are displayed as scFvs. Several human antibodies were obtained from these libraries, used alone or in combination in biopanning processes [18,19]. Another strategy is the construction of a library obtained from amplifying the variable gene repertoires of one or more individuals. For this aim, primers covering all V gene families are used and the library is generated by a random combination of VL and VH chain coding genes. Again, the antibody coding genes are assembled to be displayed as Fab or scFv antibody fragments [5]. Theoretically, each clone codes for a specific antigen-binding site, derived from the natural repertoire which is increased by an artificial domain shuffling that extrapolates the original repertoire.

The panning procedure mimics the B-cell clonal selection system *in vitro* by specifically enriching phage particles that display antibodies with a desired specificity [15]. During library construction it is important to assure library diversification, thus the size of the library is pivotal for attempting efficacy in selecting binding forms for any given antigen. Combinatorial antibody libraries can be constructed from a repertoire of immunized [20–22] or non immunized donors [20–22]. Even in the last case, selection provides high-affinity antibodies [23] against different types of antigen: haptens, proteins, peptides and carbohydrates.

**Figure 1:**
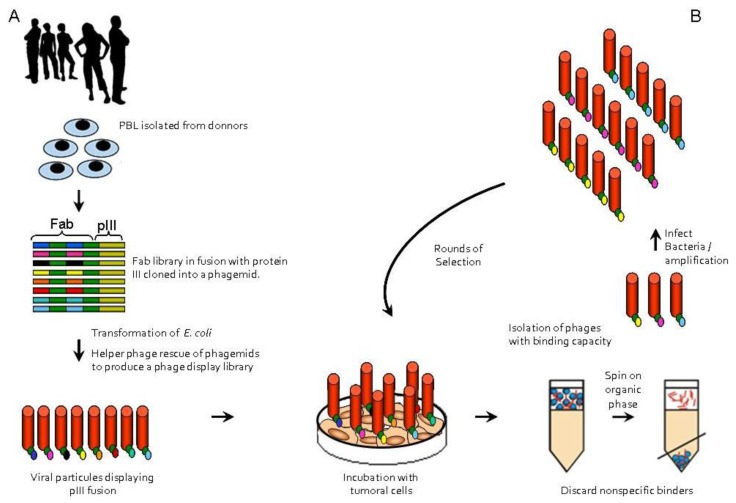
General scheme of phage display technology. (**A**) Generation of a phage display library by mRNA extraction, cDNA cloning into a phagemid vector and phage propagation; (**B**) Biopanning: affinity against tumoral cells driven enrichment of target specific binders during several rounds of selection.

Most of the panning methods are based on four major steps: preparation of phage-displayed libraries, adsorbing the specific binding phage, removal of nonspecific or low affinity phages, and recovering of target binders, that will be reamplified after bacteria infection for the next round of selection [24]. The selection procedure results in the sequential enrichment of phages displaying specific binding to a chosen pure or impure antigen (Figure 1). These panning steps will be repeated, usually 3 to 5 times, until the identification of high specific/affinity binder [25,26]. Commonly, a purified antigen is immobilized into a support like resin or beads. More complex systems such as eukaryotic cells and tissue fragments can be used and even *in vivo* panning has been reported. The utilization of a phage display library to target organs *in vivo* was first described by Pasqualini and Ruoslahti, in 1996 [27]. Automated phage display screening for high throughput antibody development, which increase reproducibility of the selection protocol, has been developed [28].

Selecting phages on the cell surface is an important step toward the development of targeted therapeutics for cancer; it opens the opportunity to obtain antibodies against unknown and non-immunogenic cell surface antigens in their native form. As the target antigen becomes more complex, such as cell surface, the selection procedure becomes more difficult. The cell membrane presents a myriad of proteins, carbohydrates and lipids as potential antigens. Their expression level can differ from cell to cell, which renders the presence of a relevant antigen to a minute amount among all the cell membrane components. As a consequence, a relatively large number of cells (10^5^–10^7^) is required for each round of selection, making the application of this technique difficult for small cell populations such as usually is obtained from organs and tissues. Efforts have been made to improve selection on the cell surface, once panning methods are based on extensive washes, turning them laborious, cell consuming and often inefficient, yielding variable results [29]. More efficient methods have been proposed, which includes centrifugation on density gradient or on organic phase instead of washing [29,30]. Recently a new cell-surface biopanning method based on the microfluidics technology was proposed. The accurate control of washing stringency in the microfluidic magnetic separator (MMS) circumvents cell loss and allows efficient removal of weakly or nonspecifically-bound phages [31]. The small numbers of cells required constitutes an advantage, rendering MMS a promising tool for the discovery of biomarkers from patient samples.

The phage display library approach has been extended to other alternatives including ribosomal [32] yeast [33], mammalian systems [34] or eukaryotic viruses [35], which have been extensively discussed elsewhere [36,37] and are not the objective of this review. A strategy called protein scaffolds proposes to engineer non-immunoglobulin proteins by the incorporation of a customized affinity domain [38]. It represents an alternative to antibodies for molecular recognition and is presented as libraries in phage display-scaffolds [38,39]. This is beyond the scope of this review since we will concentrate on the antibody phage display systems.

### 2.1. Immunized Phage Display Libraries

The construction of a phage display library using the entire antibody genes repertoire of a cancer patient followed by controlled panning procedure allows the identification of rare antibodies based on their binding specificities [15]. The utilization of immune libraries to detect tumoral cells antigens demonstrates the occurrence of an immunodominance phenomenon in certain cancers [40]. The immunodominance corresponds to the presence of a dominant antigen recognized for the majority of the immune response components. If an immunodominance phenomenon occurs, the diversity of selected clones harboring specific antibodies will be limited. The specificity of the majority population of isolated antibodies will be determined by the antigenic profile of the cells used. From melanoma patients there are two examples of limited selected antibodies using antibody libraries from patients. A fusion scFv phage library expressing the antibody repertoire of two melanoma patients immunized with cultured autologous tumor cells, transduced with the γ-interferon gene to enhance immunogenicity was constructed [41]. A Fab library also from melanoma patients was constructed by Pereira *et al*. [42]. From both libraries, only one specific clone was selected, suggesting that an immunodominant epitope may exist in melanoma cells. Immunodominance seems to occurs also in osteosarcoma, in a previous work we constructed a Fab phage display library from osteosarcoma patients, after selection on osteosarcoma cell lines we were able to isolate 5 tumor associated Fabs; despite sharing different VH and VL sequences, they recognize the same over expressed protein in the osteosarcoma cells and tissue [43]. Other examples include the selection of antibodies against anti-FBP a protein highly expressed in ovarian carcinoma [44], anti-melanoma and breast cancer [45].

The serological identification of Ags by recombinant cDNA expression cloning (SEREX) method was designed to exploit patient serum to probe a tumor specific cDNA expression library with autologous patient serum (serological cDNA screening). SEREX allowed the identification of immunogenic tumor proteins in many tumor types [46,47]. Nevertheless, this method is laborious and the nonquantitative format of the secondary screening assay on an individual serum hampers the analysis of large panels of candidate antigens against hundreds of serum samples of limited amounts. To circumvent this problem, another method allying SEREX to phage display technology was developed and called SAS (serological Ag selection). This procedure uses repeated cycles of selection and amplification of phage cDNA libraries on patient serum to enrich immunoreactive cDNA products displayed on the phage surface [48]. The SAS method allowed the identification of a panel of candidate tumor antigens in colorectal cancer [49]. Further, a robust approach combining phage display library derived from cancer tissue with protein microarray was proposed by Fernandez-Madrid *et al*. [50]. At first, a phage display cDNA library from breast cancer samples was screened against sera from 10 patients, then a microarray of the positive phages was probed with sera from another 90 patients. The selected autoantigens could significantly discriminate between breast cancer and non-cancer control sera. A similar approach has been used to characterize new autoantibody-binding peptides derived from a prostate-cancer tissue phage library. A panel of 22 potentially diagnostic markers, contained, among others, eIF4G1, a protein that is over expressed in prostate-cancer epithelial tissue [51]. Again a combination of phage display with high-density peptide microarray was used to detect the autoantibodies in the sera from lung adenocarcinoma patients. The study led to the detection of a number of novel peptide targets that elicit a humoral immune response in lung cancer patients and can predict cancer status with 85% sensitivity and 86% specificity. Among the identified peptides there was ubiquilin 1 (a protein that regulates the degradation of several ubiquitin-dependent proteasome substrates), which is significantly increased in lung tumors [52]. These studies suggest that the humoral immune response may be useful in the diagnosis and classification of tumors and pave the way of the emerging area, termed “cancer immunomics” [53] which constitutes the analysis of the host humoral immune response against tumoral cells.

### 2.2. Immunized Animals as Source of Antibody Repertoire

Immunized animals can also be used as a source of antibody repertoire. Potential therapeutic antibodies were identified from a phage library displaying antibodies from rabbits immunized with primary chronic lymphocytic leukemia (CLL) cells [54]. A first generation of chimerical rabbit/human Fab and IgG1 that bind ROR1 with high affinity and specificity was generated from those immunized rabbits and selected by phage display. This work provides both rationale and platform for a second generation of mAbs and antibody derivatives [55]. A single-chain Fv antibody fragment specific for CD123 were isolated from a phage display library generated from mice spleen mRNA. The animals were immunized with a fusion protein consisting of the extracellular domain of CD123 and the Fc domain of a human immunoglobulin. The scFv with the highest affinity for leukemia stem cells (LSCs) in acute myeloid leukemia (AML) was used to design two cell death-inducing molecules. First, an immunotoxin, a fusion protein with truncated *Pseudomonas* Exotoxin A, induced potent apoptosis of AML-derived cells. Second, a bispecific single chain Fv (bsscFv) created by the fusion to another scFv specific for CD16, which mediated potent lysis of AML-derived cells inducing antibody-dependent cellular cytotoxicity (ADCC) reactions. The recruitment of CD16-positive effectors cells for the lysis of AML cells via CD123 opens a promising combination for future clinical testing [56].

Another molecule format, the sdAb, is a very useful component in antibody engineering as potential tools for diagnostic and therapeutic application. Baral *et al.* [57] have isolated an sdAb against CEACAM 6 from a llama immunized with cancer cells. This molecule reduces proliferation on CEACAM expressing cells and shows excellent tumor targeting *in vivo*, thus presenting potential in diagnosis and therapy of CEACAM6 expressing tumors. Recently Bell *et al*. [58] described the isolation of eleven sdAbs from an imunized llama that target EGFR and the construction of pentabody (V2C-EG2) and cHCAb (EG2-hFc) versions of one of these sdAbs (EG2). These three versions of EG2 were radiolabeled with ^64^Cu and microPET/CT imaging was used to analyze their *in vivo* distribution in a human pancreatic carcinoma xenograft model. Whereas EG2 and V2C-EG2 localized mainly in the kidneys after i.v. injection, EG2-hFc exhibited excellent tumor accumulation, and this was largely attributed to its long serum half-life, which is comparable to that of IgGs. The moderate size (~80 kDa) and intact human Fc make HCAbs a unique antibody format which may outperform whole IgGs as imaging and therapeutic reagents.

These examples illustrate that, undoubtedly, phage display technologies have great potential for proteome-wide exploration of humoral immune response and for production of therapeutic antibodies, either from cancer immunized patients or animals.

### 2.3. Nom-Immune Libraries to Identify Binders to Tumor Markers

Phage display panning strategy using the universal non-immunized (*naïve*) libraries of scFv has been largely used to specifically identify binders against most important targets in oncology. Here we explore some interesting molecules obtained from naïve antibody libraries against VEGF and HER2.

Signal transduction through the vascular endothelial growth factor (VEGF) and its receptor VEGFR-2/kinase domain receptor (KDR) plays a crucial role in angiogenesis, and has therefore become a major target for therapeutic applications [59]. Phage display has been largely exploited to select high affinity binders to VEGF. Different molecule formats have been proposed such as VH binders [60], disulfide-stabilized single chain antibody variable fragments (sc-dsFv) [61], anti-VEGF ccFv (Fab-like antibody binding unit in which a pair of heterodimeric coiled-coil domains was fused to VH and VL) antibody [62]. The specific interaction between VEGF and its receptors has been targeted by blocking agents such as Fab [63], peptide [64], or scFv targeting the VEGF165 isoform [65]. Phage display was even used against mutated forms of VEGF [66], or VEGF-C [67] and *in vivo*, to identify tumor-homing peptides that specifically target tumor blood vessels, with the potential to improve the systemic treatment of patients with solid tumors [68]. We must mention also the Ramucirumab, a monoclonal antibody derived from phage display that is used in clinical trials, and will be detailed later.

Epidermal growth factor receptors (EGFRs) are overexpressed and/or dysregulated in many tumor types. The EGFR family contains four members: EGFR1 (ErbB1), HER2 (ErbB2), HER3 (ErbB3) and HER4 (ErbB4) [69]. Human Epidermal Growth Factor Receptor-2 HER2 (erbB2, HER2/neu) is highly expressed in some breast cancers, ovarian and gastric cancers [70]. Since the discovery of its role in tumorigenesis, HER2 has been largely explored as a tumoral target leading to the successful development of the humanized monoclonal anti-HER2 antibody (Trastuzumab) used for breast cancer treatment. Phage display has contributed, especially in the identification of smaller molecules. A large number of HER2 specific binders for diagnostic and therapy have been proposed. Phage display has contributed to identification of HER2 antibodies [71], peptides [72], llama anti-Id single domain antibody (sdAb), anti-HER2 [73] and human anti-Id scFv against trastuzumab [74].

## 3. Phage Display to Select Antibodies for Molecular Imaging

Medical imaging technologies have become a crucial tool in oncology. Advances provided by genetics, biochemistry, immunology and cellular biology concerning tumorigenesis need to be exploited. Molecular probes are not limited to tumor location; they became an integral part of clinical trials allowing monitoring of individualized treatment and may help drug development. Usually the majority of such probes are obtained from peptides phage libraries once peptides present high affinity and selectivity, and are also less immunogenic, showing lower toxicity and exhibiting predictable pharmacokinetic properties. Nevertheless, antibodies, mainly fragments, have also been utilized due to their intrinsic high affinity.

It is not surprisingly that the majority of antibodies selected for molecular imaging target angiogenesis. Zehnder-Fjällman *et al*. [75] selected a scFv targeting VEGFR-3. The clone AFC5 showed specific tumor accumulation in a hVEGFR-3 expressing F9 teratocarcinoma in a mouse model by combined single proton emission computed tomography (SPECT/CT) and immunohistochemical analysis. The antibody has also the ability to block the interaction of hVEGF-C to its receptor; thus, reducing proliferation of human lymphatic endothelial cells. Apart from VEGF/VEGFs, angiogenesis can be targeted in other ways, such as by integrins and MMPs and even to fibronectin, a high-molecular weight glycoprotein that binds to integrins [76]. The oncofetal fibronectin (B-FN) isoform is present in vessels of neoplastic tissues during angiogenesis but not in mature vessels. Phage display was used to isolate human antibody targeting this specific fibronectin isoform. The scFv (L19) was identified and has been shown to efficiently localize on neovasculature *in vivo*. Imaging in real time was done to study biodistribution of scFv L19 coupled chemically to a fluorophore by infrared photodetection in human teratocarcinoma bearing mice [77]. Later, the same L19 small immunoprotein (SIP) was labeled by ^76^Br allowing detailed small-animal PET of tumor neovasculature [78]. ^124^I-L19-SIP is also suitable for immuno-PET [79].

To study the vascular endothelia, a phage-displayed antibody was converted into a single-chain variable fragment and fused to the crystallizing fragment of an antibody (scFv-Fc). Such fusion proteins are able to rapidly target vascular endothelia of specific organs *in vivo*. Radioiodinated scFv-Fcs homing to specific organs were observed *in vivo* imaged by planar scintigraphy [80]. Other examples include an anti-c-Met scFv conjugated quantum that reveals higher tumor uptake and increased tumor-normal tissue ratios by *in vivo* fluorescent imaging [81]. Those molecules are valuable tools to expand our understanding of tumorigenesis.

## 4. Antibodies Derived from Phage Display Libraries Used in Clinical Trials

The most successfully derived phage display antibodies proved their efficacy in pre-clinical studies and are currently being tested in clinical trials as single agents, in combination regimens or as radiolabeled molecules for imaging. These results are presented in Table 1, and are detailed below.

### 4.1. 1D09C3 (GPC Biotech AG)

1D09C3 is an anti-MHC class II (HLA-DR) fully human IgG4 antibody directed against cell surface antigens. Antibodies of the IgG4 subclass present the advantage of poor induction of the complement system, and as a consequence have received great attention as therapeutic molecules. It is known that the major histocompatibility complex class II protein HLA-DR is highly expressed in various tumors, including T and B-cell lymphomas and in a variety of autoimmune and inflammatory diseases. 1D09C3 induces apoptosis in MHC-II positive cells [11]. A radiolabeled fully humanized IgG4 monoclonal antibody (mAb) can provide a useful tool for prognostics and diagnostics. Malviya *et al*. [82] attested the efficacy of labeled 1D09C3 with technetium-99m to target HLA-DR antigens. Successful preclinical results in lymphoma [83] and myeloma, in combination with γ-IFN [84] have been reported. 1D09C3 received the orphan drug designation for the treatment of Hodgkin’s lymphoma by the European Medicines Agency (EMEA) in 2005. Nevertheless, no conclusive result on phase I clinical trials are published so far.

### 4.2. Fresolimumab (GC-1008—Cambridge Antibody Technology/Genzyme)

Fresolimumab is a human IgG4 monoclonal antibody that neutralizes all three isoforms of transforming growth factor-β. TGF-β is involved in a variety of cellular processes such as proliferation, differentiation, and migration and is overexpressed in many tumor cells. Suppression of TGF-β using antibodies, soluble receptors or inhibitors of TGF-β signal transduction has shown beneficial effects in murine tumor models. The G4 immunoglobulin (IgG4) subclass does not activate the complement pathway, a suitable characteristic for an antibody. Fresolimumab tumor uptake and organ distribution could be visualized and quantified with 89Zr-fresolimumab PET in tumor models [85]. This technique could be a valuable tool to identify patients who would benefit most from such an anti-cancer treatment. A phase II study to evaluate 89Zr-GC1008 PET uptake in malignant gliomas (NCT01472731) is ongoing along with a clinical trial to evaluate fresolimumab and radiotherapy in metastatic breast cancer (NCT01401062).

### 4.3. Cixutumumab (IMC-A12—ImClone Systems Incorporated)

Cixutumumab is a fully human immunoglobulin G1 (IgG1) monoclonal antibody that selectively binds to membrane-bound IGF-1R (insulin like growth factor-1 receptor) thereby preventing the binding of the natural ligand IGF-1 (insulin-like growth factor). The high affinity of cixutumumab (kd = 10^−11^ M) is sufficient to block ligand induced receptor activation. IMC-A12 inhibited downstream signaling of the two major IGF pathways, mitogen-activated protein kinase and phosphatidylinositol 3′-kinase/Akt, but does not block the insulin receptor. A marked increase in apoptotic tumor cells was observed in *in vivo* tumor models [86]. Promising preclinical results with IMC-A12 suggested that it may be useful in effective therapeutic for a diverse array of oncologic indications [87]. There are 32 Phase I–II ongoing clinical trials employing cixutumumab. The first published data revealed low toxicity and moderate effects. In the phase I/II study in advanced non-small cell lung cancer (NSCLC), cixutumumab was tolerated as a single agent but not in combination with erlotinib; nevertheless, the efficacy in unselected patients with NSCLC seemed to be low [88]. A phase II study in patients with cetuximab or panitumumab-refractory metastatic colorectal cancer showed that IMC-A12 alone or in combination with cetuximab was insufficient to warrant additional study in patients with colorectal cancer refractory to EGFR inhibitors [89]. Moreover, cixutumumab was well tolerated in children with refractory Ewing sarcoma and a phase II was recommended, even limited single-agent activity observation [90].

### 4.4. Necitumumab (IMC-11F8—ImClone Systems Incorporated)

Necitumumab is a fully human IgG1 monoclonal antibody targeting the epidermal growth factor receptor (EGFR). Mutations that activate EGFR are present in diverse tumors including colorectal and non small-cell lung cancer (NSCLC). Several EGFR inhibitors like erlotinib and gefinitib are approved as anticancer agents in the US and Europe [91]. Necitumumab binds to the EGFR with high affinity (kd = 0.32 nmol/L), and blocks the binding of EGFR ligands, neutralizing ligand-induced EGFR phosphorylation. As a consequence, necitumumab inhibits downstream targets in the EGFR pathway (e.g., MAPK) inhibiting proliferation of EGFR-dependent tumor cells. Moreover, it has the potential to induce antibody-dependent cell-mediated cytotoxicity against tumor cells [92]. Preclinical studies indicated that the antitumor activity of necitumumab is either comparable with or superior to that of Cetuximab, having the potential benefit of lower hypersensitivity reaction risk. There is two phase I clinical trials in solid tumors (NCT01088464, NCT00801177) and a phase II in colorectal cancer combined with 5-FU/FA and Oxaliplatin ongoing (NCT00835185). Necitumumab toxicity is acceptable; with skin toxicity being the most frequently reported adverse event in the clinical trial studies [93]. Recently, a Phase III study of necitumumab combined with pemetrexed-cisplatin chemotherapy (INSPIRE) in patients with stage IV nonsquamous NSCLC was prematurely closed due to increased risk of thromboembolic events in the experimental arm [94]. Nevertheless, another Phase III trial of necitumumab in combination with gemcitabine and cisplatin in squamous NSCLC is ongoing (NCT00981058). Success in this study will determine the clinical significance of this drug to future therapeutic strategies in NSCLC.

### 4.5. Ramucirumab (IMC-1121B—ImClone Systems Incorporated)

Ramucirumab (IMC-1121B) is a fully human IgG1 monoclonal antibody targeting the vascular endothelial growth factor receptor-2 (VEGFR-2). Similar to bevacizumab (or Avastin, a FDA approved antibody), which directly binds to and neutralizes circulating VEGF [95], ramucirumab targets angiogenesis, an essential tumor-stroma interaction [96]. IMC-1121B’s unique mechanism of action specifically blocks VEGFR-2 activation, allowing for maximal angiogenesis inhibition. Ramucirumab is well tolerated, the most frequent related dose-limiting toxicities are hypertension and deep venous thrombosis, nausea, vomiting, headache, fatigue, and proteinuria were also noted [97]. Ramucirumab is under evaluation in 27 phase I–III clinical trials in a broad array of cancers. A multinational randomized phase III trial will evaluate the efficacy and safety of ramucirumab, as standard first-line docetaxel chemotherapy for women with HER2-negative metastatic breast cancer by means of progression-free survival and overall survival [98]. Another Phase III study evaluating the safety and efficacy of ramucirumab as 2^nd^-line treatment in patients with hepatocellular carcinoma after 1^st^-line therapy with Sorafenib is under way (NCT01140347).

### 4.6. Mapatumumab (HGS-ETR1—Human Genome Sciences Inc./GlaxoSmithKline)

Mapatumumab (HGS-ETR1) is a human monoclonal antibody that specifically binds to TRAIL receptor-1 inducing cancer-cell death by apoptosis. TRAIL induces apoptosis through activation of death receptors TRAIL-R1 and TRAIL-R2. TRAIL receptor 1 is expressed on a variety of tumors. Mapatumumab has agonist activity and demonstrates tumor cell killing by apoptosis *in vitro* in a broad array of human cancer types and in preclinical animal models [99,100]. Mapatumumab was efficiently radiolabeled with 111In and can be used clinically to study pharmacokinetics, biodistribution and tumor targeting, which could support evaluation of the native targeted agents in phase I/II trials [101]. Results from phase I and II studies have been reported and showed that Mapatumumab has potential either as a single agent or in combination with chemotherapy for the treatment of cancer. A phase Ib/II trial in patients with relapsed non-Hodgkin’s lymphoma (NHL) proved that mapatumumab is well tolerated, no patients experiencing drug-related hepatic or other dose-limiting toxicity. Results from three patients with follicular lymphoma (FL) showed a complete clinical response for two of them and a partial response in the third one. This places mapatumumab as a promising new therapeutic for patients with FL [102]. In a Phase II clinical trial in patients with refractory colorectal cancer Mapatumumab showed no clinical activity as single-agent, however, a potential synergy in combination with agents commonly used in the treatment of colorectal cancer warrants further evaluation [103]. A phase I study of mapatumumab in combination with Nexavar (sorafenib) is currently underway in patients with advanced hepatocellular carcinoma (NCT00712855) and a Phase II study of mapatumumab in combination with bortezomib and bortezomib alone in subjects with relapsed or refractory multiple myeloma (MM) is ongoing (NCT00315757).

### 4.7. Lexatumumab (HGS-ETR2—Human Genome Sciences Inc.)

Lexatumumab (HGS-ETR2) is a fully human agonistic mAb that targets the tumor necrosis factor-related apoptosis-inducing ligand receptor 2 (TRAIL-R2). Lexatumumab activates the extrinsic apoptosis pathway. Upon binding to TRAIL-R2, HGS-ETR2 forms a death-inducing signaling complex with the adaptor protein FAS-associated death domain. Following, there is activation of caspases 8 or 10, which then activate caspases 3, 6, and 7, resulting in apoptosis [104]. Lexatumumab showed potent preclinical antitumor activity mainly to solid tumors. A phase I study performed in 37 patients with advanced solid tumors showed that lexatumumab was well tolerated and warrants additional evaluation, particularly in combination with chemotherapy agents [105]. In another phase I study, despite the observation that one patient experienced a possibly related dose-limiting toxicity of grade 3 hyperamylasemia, they concluded that lexatumumab can be safely administered. Further evaluation of lexatumumab alone or in combination with other agents is suitable [106]. A phase I trial of lexatumumab with or without interferon gamma in patients with refractory pediatric solid tumors is underway (NCT00428272).

### 4.8. Moxetumomab Pasudotox (HA22, CAT-8015, (RFB4(dsFv)-PE38—MedImmune)

CAT-8015 is an anti-CD22 immunotoxin fusion protein between a murine anti-CD22 disulfide-linked Fv (dsFv) antibody fragment and a *Pseudomonas* exotoxin PE38. The original molecule CAT-3888 (BL22), consisted of disulfide linked VH and VL chains of the mouse anti-CD22 monoclonal antibody RFB4 fused to a truncated form of ETA, PE38. RFB4 was originated from a mouse hybridoma [107] although phage display was employed to improve this molecule. CAT-8015 is a second-generation CD22 targeted immunotoxin. The PE38 portion of this construct is identical to that used for CAT-3888 but the VH and VL chains have been affinity matured by phage display from a library targeting the CDR3 domain of the VH chain in a scFv format [108]. This procedure resulted in a variant containing three amino acids changes in the CDR3 that increased binding affinity for the target molecule (approximately 14-fold, kd approximately 6 nM) when compared to the parental protein. The Fv binds to an antigen on a target cell and brings the toxin into the cell interior, where it arrests protein synthesis and initiates the apoptotic cascade [109]. CAT-8015 has greatly improved efficacy compared with CAT-3888 in preclinical studies using B-cell *in vitro* and *in vivo* tumor models [110]. There are 8 phase I/II clinical trials using CAT-8015 for leukemia or lymphomas. Recently a phase I dose-escalation trial in chemotherapy-resistant hairy cell leukemia (HCL) was published. No dose-limiting toxicity was observed among the 28 patients enrolled [111].

## 5. Conclusions

Combinatorial antibody phage display technologies provide robust means for the rapid discovery of tumor antigen antibodies [13]. It is easy to implement and inexpensive. Numerous academic and industrial laboratories have adopted the technique and contributed to its advancement. The methods for design, construction and screening of synthetic antibody libraries are constantly being improved and semi-automated selection strategies have been proposed. The advantage of antibody phage display is the direct isolation of fully human antibodies in comparison to antibodies generated by mouse hybridoma technology where a laborious humanization procedure of the lead candidates is required. Many products of phage display are now reaching the Biopharmaceutical market. Monoclonal antibodies had been the standard as anticancer agents by modulating intrinsic tumor cell pathways and activating the innate immune response against cancer cells. Phage display derived human antibodies obviously can compete with earlier generation products as long as they are directed to well defined targets such as the EGFR or VEGF pathway. Phage display plays an important role in drug discovery and development with a number of molecules approved by FDA and many others in clinical trials. It is normal to expect that greater improvement will be made in the near future and phage display will bring many more contributions to oncology.

## Figures and Tables

**Table 1:** Examples of monoclonal antibodies derived from phage display technology currently under evaluation for cancer therapy in clinical trials. Phage display monoclonal antibodies used in clinical trials.

Name	Target	Format	Phase	Indication
1D09C3	HLA-DR	H IgG4	I	Hodgkin’s lymphoma, myeloma
Lexatumumab	TRAIL-R2	H IgG1	I	Advanced solid tumors
*Moxetumomab pasudotox*	CD22	Recombinant immunotoxin	I,II	Acute lymphoblastic leukemia, hairy cell leukemia, non-Hodgkin lymphoma
Fresolimumab	TGFβ	H IgG4	I,II	Breast cancer; gliomas, kidney cancer; melanoma; mesothelioma.
Cixutumumab	IGF1R	H IgG1	I,II	Adrenocortical carcinoma; breast cancer; colorectal cancer; HNC; islet cell cancer; liver cancer; Malignant Fibrous Histiocytoma neuroendocrine cancer; NSCLC; pancreatic cancer; prostate cancer; sarcoma; SCLC; solid tumors.
Mapatumumab	TRAIL-R1	H IgG1	II	Advanced Cervical Cancer, Hepatocellular Carcinoma, Multiple Myeloma
Necitumumab	EGFR	H IgG1	III	NSCLC
Ramucirumab	VEGFR2	H IgG1	III	Hepatocellular carcinoma, metastatic gastric or gastresophageal junction adenocarcinoma

## References

[1] Berger M., Shankar V., Vafai A. (2002). Therapeutic applications of monoclonal antibodies. Am. J. Med. Sci.

[2] Seymour J.F. (2004). New treatment approaches to indolent non-Hodgkin’s lymphoma. Semin. Oncol.

[3] Cosimi A.B., Colvin R.B., Burton R.C., Rubin R.H., Goldstein G., Kung P.C., Hansen W.P., Delmonico F.L., Russell P.S. (1981). Use of monoclonal antibodies to T-cell subsets for immunologic monitoring and treatment in recipients of renal allografts. N. Engl. J. Med.

[4] Kohler G., Milstein C. (1975). Continuous cultures of fused cells secreting antibody of predefined specificity. Nature.

[5] Maranhao A.Q., Brigido M.M. (2000). Expression of anti-Z-DNA single chain antibody variable fragment on the filamentous phage surface. Braz. J. Med. Biol. Res.

[6] Morrison S.L., Oi V.T. (1989). Genetically engineered antibody molecules. Adv. Immunol.

[7] Holliger P., Hudson P.J. (2005). Engineered antibody fragments and the rise of single domains. Nat. Biotechnol.

[8] Holt L.J., Herring C., Jespers L.S., Woolven B.P., Tomlinson I.M. (2003). Domain antibodies: Proteins for therapy. Trend. Biotechnol.

[9] Hamers-Casterman C., Atarhouch T., Muyldermans S., Robinson G., Hamers C., Songa E.B., Bendahman N., Hamers R. (1993). Naturally occurring antibodies devoid of light chains. Nature.

[10] McCafferty J., Griffiths A.D., Winter G., Chiswell D.J. (1990). Phage antibodies: Filamentous phage displaying antibody variable domains. Nature.

[11] Thie H., Meyer T., Schirrmann T., Hust M., Dubel S. (2008). Phage display derived therapeutic antibodies. Curr. Pharm. Biotechnol.

[12] ClinicalTrials.gov Home Page.

[13] Smith G.P. (1985). Filamentous fusion phage: Novel expression vectors that display cloned antigens on the virion surface. Science.

[14] Posner B., Smiley J., Lee I., Benkovic S. (1994). Catalytic antibodies: Perusing combinatorial libraries. Trends Biochem. Sci.

[15] Barbas C.F., Kang A.S., Lerner R.A., Benkovic S.J. (1991). Assembly of combinatorial antibody libraries on phage surfaces: The gene III site. Proc. Natl. Acad. Sci. USA.

[16] Clackson T., Hoogenboom H.R., Griffiths A.D., Winter G. (1991). Making antibody fragments using phage display libraries. Nature.

[17] Tomlinson Library Home Page.

[18] Chinestra P., Lajoie-Mazenc I., Faye J.C., Favre G. (2012). Use of phage display for the identification of molecular sensors specific for activated Rho. Meth. Mol. Biol.

[19] Davern S.M., Foote L.J., Lankford T.K., Macy S.D., Wall M.D., Kennel S.J. (2005). Identification of an antilaminin-1 scFv that preferentially homes to vascular solid tumors. Cancer Biother. Radiopharm.

[20] Barbas C.F., Burton D.R. (1996). Selection and evolution of high-affinity human anti-viral antibodies. Trends Biotechnol.

[21] Dantas-Barbosa C., Brigido M.M., Maranhao A.Q. (2005). Construction of a human Fab phage display library from antibody repertoires of osteosarcoma patients. Genet. Mol. Res.

[22] Portolano S., McLachlan S.M., Rapoport B. (1993). High affinity, thyroid-specific human autoantibodies displayed on the surface of filamentous phage use V genes similar to other autoantibodies. J. Immunol.

[23] Griffiths A.D., Malmqvist M., Marks J.D., Bye J.M., Embleton M.J., McCafferty J., Baier M., Holliger K.P., Gorick B.D., Hughes-Jones N.C. (1993). Human anti-self antibodies with high specificity from phage display libraries. EMBO J.

[24] Mandecki W., Chen Y.C., Grihalde N. (1995). A mathematical model for biopanning (affinity selection) using peptide libraries on filamentous phage. J. Theor. Biol.

[25] Marks J.D., Hoogenboom H.R., Bonnert T.P., McCafferty J., Griffiths A.D., Winter G. (1991). By-passing immunization. Human antibodies from V-gene libraries displayed on phage. J. Mol. Biol.

[26] Vaughan T.J., Williams A.J., Pritchard K., Osbourn J.K., Pope A.R., Earnshaw J.C., McCafferty J., Hodits R.A., Wilton J., Johnson K.S. (1996). Human antibodies with sub-nanomolar affinities isolated from a large non-immunized phage display library. Nat. Biotechnol.

[27] Pasqualini R., Ruoslahti E. (1996). Organ targeting *in vivo* using phage display peptide libraries. Nature.

[28] Glokler J., Schutze T., Konthur Z. (2010). Automation in the high-throughput selection of random combinatorial libraries—Different approaches for select applications. Molecules.

[29] Williams B.R., Sharon J. (2002). Polyclonal anti-colorectal cancer Fab phage display library selected in one round using density gradient centrifugation to separate antigen-bound and free phage. Immunol. Lett.

[30] Giordano R.J., Cardo-Vila M., Lahdenranta J., Pasqualini R., Arap W. (2001). Biopanning and rapid analysis of selective interactive ligands. Nat. Med.

[31] Liu Y., Adams J.D., Turner K., Cochran F.V., Gambhir S.S., Soh H.T. (2009). Controlling the selection stringency of phage display using a microfluidic device. Lab Chip.

[32] He M., Taussig M.J. (1997). Antibody-ribosome-mRNA (ARM) complexes as efficient selection particles for *in vitro* display and evolution of antibody combining sites. Nucleic Acids Res.

[33] Wang X.X., Shusta E.V. (2005). The use of scFv-displaying yeast in mammalian cell surface selections. J. Immunol. Meth.

[34] Amstutz P., Forrer P., Zahnd C., Pluckthun A. (2001). *In vitro* display technologies: Novel developments and applications. Curr. Opin. Biotechnol.

[35] Michelfelder S., Lee M.K., deLima-Hahn E., Wilmes T., Kaul F., Muller O., Kleinschmidt J.A., Trepel M. (2007). Vectors selected from adeno-associated viral display peptide libraries for leukemia cell-targeted cytotoxic gene therapy. Exp. Hematol.

[36] Grabherr R., Ernst W. (2010). Baculovirus for eukaryotic protein display. Curr. Gene Ther.

[37] Pepper L.R., Cho Y.K., Boder E.T., Shusta E.V. (2008). A decade of yeast surface display technology: Where are we now?. Comb. Chem. High Throughput Screen.

[38] Skerra A. (2007). Alternative non-antibody scaffolds for molecular recognition. Curr. Opin. Biotechnol.

[39] Zoller F., Haberkorn U., Mier W. (2011). Miniproteins as phage display-scaffolds for clinical applications. Molecules.

[40] Wortzel R.D., Urban J.L., Philipps C., Fitch F.W., Schreiber H. (1983). Independent immunodominant and immunorecessive tumor-specific antigens on a malignant tumor: Antigenic dissection with cytolytic T cell clones. J. Immunol.

[41] Cai X., Garen A. (1995). Anti-melanoma antibodies from melanoma patients immunized with genetically modified autologous tumor cells: Selection of specific antibodies from single-chain Fv fusion phage libraries. Proc. Natl. Acad. Sci. USA.

[42] Pereira S., Maruyama H., Siegel D., van Belle P., Elder D., Curtis P., Herlyn D. (1997). A model system for detection and isolation of a tumor cell surface antigen using antibody phage display. J. Immunol. Meth.

[43] Dantas-Barbosa C., Faria F.P., Brigido M.M., Maranhao A.Q. (2009). Isolation of osteosarcoma-associated human antibodies from a combinatorial Fab phage display library. J. Biomed. Biotechnol.

[44] Figini M., Obici L., Mezzanzanica D., Griffiths A., Colnaghi M.I., Winter G., Canevari S. (1998). Panning phage antibody libraries on cells: Isolation of human Fab fragments against ovarian carcinoma using guided selection. Cancer Res.

[45] Lee K.J., Mao S., Sun C., Gao C., Blixt O., Arrues S., Hom L.G., Kaufmann G.F., Hoffman T.Z., Coyle A.R. (2002). Phage-display selection of a human single-chain fv antibody highly specific for melanoma and breast cancer cells using a chemoenzymatically synthesized G(M3)-carbohydrate antigen. J. Am. Chem. Soc.

[46] Sahin U., Tureci O., Schmitt H., Cochlovius B., Johannes T., Schmits R., Stenner F., Luo G., Schobert I., Pfreundschuh M. (1995). Human neoplasms elicit multiple specific immune responses in the autologous host. Proc. Natl. Acad. Sci. USA.

[47] Scanlan M.J., Gordan J.D., Williamson B., Stockert E., Bander N.H., Jongeneel V., Gure A.O., Jager D., Jager E., Knuth A. (1999). Antigens recognized by autologous antibody in patients with renal-cell carcinoma. Int. J. Cancer.

[48] Hufton S.E., Moerkerk P., de Bruine A., Arends J.W., Hoogenboom H.R. (1998). Serological antigen selection of phage displayed colorectal tumour cDNA libraries. Biochem. Soc.Trans.

[49] Somers V.A., Brandwijk R.J., Joosten B., Moerkerk P.T., Arends J.W., Menheere P., Pieterse W.O., Claessen A., Scheper R.J., Hoogenboom H.R. (2002). A panel of candidate tumor antigens in colorectal cancer revealed by the serological selection of a phage displayed cDNA expression library. J. Immunol.

[50] Fernandez-Madrid F., Tang N., Alansari H., Granda J.L., Tait L., Amirikia K.C., Moroianu M., Wang X., Karvonen R.L. (2004). Autoantibodies to annexin XI-A and other autoantigens in the diagnosis of breast cancer. Cancer Res.

[51] Wang X., Yu J., Sreekumar A., Varambally S., Shen R., Giacherio D., Mehra R., Montie J.E., Pienta K.J., Sanda M.G. (2005). Autoantibody signatures in prostate cancer. N. Engl. J. Med.

[52] Chen G., Wang X., Yu J., Varambally S., Yu J., Thomas D.G., Lin M.Y., Vishnu P., Wang Z., Wang R. (2007). Autoantibody profiles reveal ubiquilin 1 as a humoral immune response target in lung adenocarcinoma. Cancer Res.

[53] Lee S.Y., Obata Y., Yoshida M., Stockert E., Williamson B., Jungbluth A.A., Chen Y.T., Old L.J., Scanlan M.J. (2003). Immunomic analysis of human sarcoma. Proc. Natl. Acad. Sci. USA.

[54] McWhirter J.R., Kretz-Rommel A., Saven A., Maruyama T., Potter K.N., Mockridge C.I., Ravey E.P., Qin F., Bowdish K.S. (2006). Antibodies selected from combinatorial libraries block a tumor antigen that plays a key role in immunomodulation. Proc. Natl. Acad. Sci. USA.

[55] Yang J., Baskar S., Kwong K.Y., Kennedy M.G., Wiestner A., Rader C. (2011). Therapeutic potential and challenges of targeting receptor tyrosine kinase ROR1 with monoclonal antibodies in B-cell malignancies. PLoS One.

[56] Stein C., Kellner C., Kugler M., Reiff N., Mentz K., Schwenkert M., Stockmeyer B., Mackensen A., Fey G.H. (2010). Novel conjugates of single-chain Fv antibody fragments specific for stem cell antigen CD123 mediate potent death of acute myeloid leukaemia cells. Br. J. Haematol.

[57] Baral T.N., Murad Y., Nguyen T.D., Iqbal U., Zhang J. (2011). Isolation of functional single domain antibody by whole cell immunization: Implications for cancer treatment. J. Immunol. Meth.

[58] Bell A., Wang Z.J., Arbabi-Ghahroudi M., Chang T.A., Durocher Y., Trojahn U., Baardsnes J., Jaramillo M.L., Li S., Baral T.N. (2010). Differential tumor-targeting abilities of three single-domain antibody formats. Cancer Lett.

[59] Klagsbrun M., D’Amore P.A. (1996). Vascular endothelial growth factor and its receptors. Cytokine Growth Factor Rev.

[60] Kastelic D., Frkovic-Grazio S., Baty D., Truan G., Komel R., Pompon D. (2009). A single-step procedure of recombinant library construction for the selection of efficiently produced llama VH binders directed against cancer markers. J. Immunol. Meth.

[61] Huang Y.J., Chen I.C., Yu C.M., Lee Y.C., Hsu H.J., Ching A.T., Chang H.J., Yang A.S. (2010). Engineering anti-vascular endothelial growth factor single chain disulfide-stabilized antibody variable fragments (sc-dsFv) with phage-displayed sc-dsFv libraries. J. Biol. Chem.

[62] Wang X., Zhong P., Luo P.P., Wang K.C. (2011). Antibody engineering using phage display with a coiled-coil heterodimeric Fv antibody fragment. PLoS One.

[63] Lu D., Jimenez X., Zhang H., Bohlen P., Witte L., Zhu Z. (2002). Selection of high affinity human neutralizing antibodies to VEGFR2 from a large antibody phage display library for antiangiogenesis therapy. Int. J. Cancer.

[64] Hetian L., Ping A., Shumei S., Xiaoying L., Luowen H., Jian W., Lin M., Meisheng L., Junshan Y., Chengchao S. (2002). A novel peptide isolated from a phage display library inhibits tumor growth and metastasis by blocking the binding of vascular endothelial growth factor to its kinase domain receptor. J. Biol. Chem.

[65] Lin Z., Cao P., Lei H. (2008). Identification of a neutralizing scFv binding to human vascular endothelial growth factor 165 (VEGF165) using a phage display antibody library. Appl. Biochem. Biotechnol.

[66] Lamdan H., Ayala M., Rojas G., Munoz Y., Morera Y., Guirola O., Chinea G., Gavilondo J.V. (2011). Isolation of a novel neutralizing antibody fragment against human vascular endothelial growth factor from a phage-displayed human antibody repertoire using an epitope disturbing strategy. J. Biotechnol.

[67] Rinderknecht M., Villa A., Ballmer-Hofer K., Neri D., Detmar M. (2010). Phage-derived fully human monoclonal antibody fragments to human vascular endothelial growth factor-C block its interaction with VEGF receptor-2 and 3. PLoS One.

[68] Chang D.K., Chiu C.Y., Kuo S.Y., Lin W.C., Lo A., Wang Y.P., Li P.C., Wu H.C. (2009). Antiangiogenic targeting liposomes increase therapeutic efficacy for solid tumors. J. Biol. Chem.

[69] Carpenter G. (1987). Receptors for epidermal growth factor and other polypeptide mitogens. Annu. Rev. Biochem.

[70] Slamon D.J., Clark G.M., Wong S.G., Levin W.J., Ullrich A., McGuire W.L. (1987). Human breast cancer: Correlation of relapse and survival with amplification of the HER-2/neu oncogene. Science.

[71] Yip Y.L., Smith G., Koch J., Dubel S., Ward R.L. (2001). Identification of epitope regions recognized by tumor inhibitory and stimulatory anti-ErbB-2 monoclonal antibodies: Implications for vaccine design. J. Immunol.

[72] Jasinska J., Wagner S., Radauer C., Sedivy R., Brodowicz T., Wiltschke C., Breiteneder H., Pehamberger H., Scheiner O., Wiedermann U. (2003). Inhibition of tumor cell growth by antibodies induced after vaccination with peptides derived from the extracellular domain of Her-2/neu. Int. J. Cancer.

[73] Alvarez-Rueda N., Ladjemi M.Z., Behar G., Corgnac S., Pugniere M., Roquet F., Bascoul-Mollevi C., Baty D., Pelegrin A., Navarro-Teulon I. (2009). A llama single domain anti-idiotypic antibody mimicking HER2 as a vaccine: Immunogenicity and efficacy. Vaccine.

[74] Coelho M., Gauthier P., Pugniere M., Roquet F., Pelegrin A., Navarro-Teulon I. (2004). Isolation and characterisation of a human anti-idiotypic scFv used as a surrogate tumour antigen to elicit an anti-HER-2/neu humoral response in mice. Br. J. Cancer.

[75] Zehnder-Fjallman A.H., Marty C., Halin C., Hohn A., Schibli R., Ballmer-Hofer K., Schwendener R.A. (2007). Evaluation of anti-VEGFR-3 specific scFv antibodies as potential therapeutic and diagnostic tools for tumor lymph-angiogenesis. Oncol. Rep.

[76] Pankov R., Yamada K.M. (2002). Fibronectin at a glance. J. Cell Sci.

[77] Neri D., Carnemolla B., Nissim A., Leprini A., Querze G., Balza E., Pini A., Tarli L., Halin C., Neri P. (1997). Targeting by affinity-matured recombinant antibody fragments of an angiogenesis associated fibronectin isoform. Nat. Biotechnol.

[78] Rossin R., Berndorff D., Friebe M., Dinkelborg L.M., Welch M.J. (2007). Small-animal PET of tumor angiogenesis using a (76)Br-labeled human recombinant antibody fragment to the ED-B domain of fibronectin. J. Nucl. Med.

[79] Tijink B.M., Perk L.R., Budde M., Stigter-van Walsum M., Visser G.W., Kloet R.W., Dinkelborg L.M., Leemans C.R., Neri D., van Dongen G.A. (2009). (124)I-L19-SIP for immuno-PET imaging of tumour vasculature and guidance of (131)I-L19-SIP radioimmunotherapy. Eur. J. Nucl. Med. Mol. Imag.

[80] Valadon P., Garnett J.D., Testa J.E., Bauerle M., Oh P., Schnitzer J.E. (2006). Screening phage display libraries for organ-specific vascular immunotargeting *in vivo*. Proc. Natl. Acad. Sci. USA.

[81] Lu R.M., Chang Y.L., Chen M.S., Wu H.C. (2011). Single chain anti-c-Met antibody conjugated nanoparticles for *in vivo* tumor-targeted imaging and drug delivery. Biomaterials.

[82] Malviya G., de Vries E.F., Dierckx R.A., Signore A. (2011). Synthesis and evaluation of 99mTc-labelled monoclonal antibody 1D09C3 for molecular imaging of major histocompatibility complex class II protein expression. Mol. Imag. Biol.

[83] Carlo-Stella C., Di Nicola M., Turco M.C., Cleris L., Lavazza C., Longoni P., Milanesi M., Magni M., Ammirante M., Leone A. (2006). The anti-human leukocyte antigen-DR monoclonal antibody 1D09C3 activates the mitochondrial cell death pathway and exerts a potent antitumor activity in lymphoma-bearing nonobese diabetic/severe combined immunodeficient mice. Cancer Res.

[84] Carlo-Stella C., Guidetti A., Di Nicola M., Lavazza C., Cleris L., Sia D., Longoni P., Milanesi M., Magni M., Nagy Z. (2007). IFN-gamma enhances the antimyeloma activity of the fully human anti-human leukocyte antigen-DR monoclonal antibody 1D09C3. Cancer Res.

[85] Oude Munnink T.H., Arjaans M.E., Timmer-Bosscha H., Schroder C.P., Hesselink J.W., Vedelaar S.R., Walenkamp A.M., Reiss M., Gregory R.C., Lub-de Hooge M.N. (2011). PET with the 89Zr-labeled transforming growth factor-beta antibody fresolimumab in tumor models. J. Nucl. Med.

[86] Burtrum D., Zhu Z., Lu D., Anderson D.M., Prewett M., Pereira D.S., Bassi R., Abdullah R., Hooper A.T., Koo H. (2003). A fully human monoclonal antibody to the insulin-like growth factor I receptor blocks ligand-dependent signaling and inhibits human tumor growth *in vivo*. Cancer Res.

[87] Rowinsky E.K., Youssoufian H., Tonra J.R., Solomon P., Burtrum D., Ludwig D.L. (2007). IMC-A12, a human IgG1 monoclonal antibody to the insulin-like growth factor I receptor. Clin. Cancer Res.

[88] Weickhardt A., Doebele R., Oton A., Lettieri J., Maxson D., Reynolds M., Brown A., Jackson M.K., Dy G., Adjei A. (2012). A phase I/II study of erlotinib in combination with the anti-insulin-like growth factor-1 receptor monoclonal antibody IMC-A12 (cixutumumab) in patients with advanced non-small cell lung cancer. J. Thorac. Oncol.

[89] Reidy D.L., Vakiani E., Fakih M.G., Saif M.W., Hecht J.R., Goodman-Davis N., Hollywood E., Shia J., Schwartz J., Chandrawansa K. (2010). Randomized, phase II study of the insulin-like growth factor-1 receptor inhibitor IMC-A12, with or without cetuximab, in patients with cetuximab- or panitumumab-refractory metastatic colorectal cancer. J. Clin. Oncol.

[90] Malempati S., Weigel B., Ingle A.M., Ahern C.H., Carroll J.M., Roberts C.T., Reid J.M., Schmechel S., Voss S.D., Cho S.Y. (2012). Phase I/II trial and pharmacokinetic study of cixutumumab in pediatric patients with refractory solid tumors and ewing sarcoma: A report from the children’s oncology group. J. Clin. Oncol.

[91] Vecchione L., Jacobs B., Normanno N., Ciardiello F., Tejpar S. (2011). EGFR-targeted therapy. Exp. Cell Res.

[92] Dienstmann R., Tabernero J. (2010). Necitumumab, a fully human IgG1 mAb directed against the EGFR for the potential treatment of cancer. Cur. Opin. Investig. Drugs.

[93] Kuenen B., Witteveen P.O., Ruijter R., Giaccone G., Dontabhaktuni A., Fox F., Katz T., Youssoufian H., Zhu J., Rowinsky E.K. (2010). A phase I pharmacologic study of necitumumab (IMC-11F8), a fully human IgG1 monoclonal antibody directed against EGFR in patients with advanced solid malignancies. Clin. Cancer Res.

[94] Dienstmann R., Felip E. (2011). Necitumumab in the treatment of advanced non-small cell lung cancer: Translation from preclinical to clinical development. Expert Opin. Biol. Ther.

[95] Ferrara N., Hillan K.J., Gerber H.P., Novotny W. (2004). Discovery and development of bevacizumab, an anti-VEGF antibody for treating cancer. Nat. Rev. Drug Discov.

[96] Hsu J.Y., Wakelee H.A. (2009). Monoclonal antibodies targeting vascular endothelial growth factor: Current status and future challenges in cancer therapy. BioDrugs.

[97] Spratlin J.L., Cohen R.B., Eadens M., Gore L., Camidge D.R., Diab S., Leong S., O’Bryant C., Chow L.Q., Serkova N.J. (2010). Phase I pharmacologic and biologic study of ramucirumab (IMC-1121B), a fully human immunoglobulin G1 monoclonal antibody targeting the vascular endothelial growth factor receptor-2. J. Clin. Oncol.

[98] Mackey J., Gelmon K., Martin M., McCarthy N., Pinter T., Rupin M., Youssoufian H. (2009). TRIO-012: A multicenter, multinational, randomized, double-blind phase III study of IMC-1121B plus docetaxel versus placebo plus docetaxel in previously untreated patients with HER2-negative, unresectable, locally recurrent or metastatic breast cancer. Clin. Breast Cancer.

[99] Menoret E., Gomez-Bougie P., Geffroy-Luseau A., Daniels S., Moreau P., Le Gouill S., Harousseau J.L., Bataille R., Amiot M., Pellat-Deceunynck C. (2006). Mcl-1L cleavage is involved in TRAIL-R1- and TRAIL-R2-mediated apoptosis induced by HGS-ETR1 and HGS-ETR2 human mAbs in myeloma cells. Blood.

[100] Pukac L., Kanakaraj P., Humphreys R., Alderson R., Bloom M., Sung C., Riccobene T., Johnson R., Fiscella M., Mahoney A. (2005). HGS-ETR1, a fully human TRAIL-receptor 1 monoclonal antibody, induces cell death in multiple tumour types *in vitro* and *in vivo*. Br. J. Cancer.

[101] Duiker E., Dijkers E., Heerspink H.L., de Jong S., van der Zee A., Jager P., Kosterink J., de Vries E., Hooge M.L. (2011). Development of a radioiodinated apoptosis-inducing ligand, rhTRAIL, and a radiolabelled agonist TRAIL receptor antibody for clinical imaging studies. Br. J. Pharmacol.

[102] Younes A., Vose J.M., Zelenetz A.D., Smith M.R., Burris H.A., Ansell S.M., Klein J., Halpern W., Miceli R., Kumm E. (2010). A Phase 1b/2 trial of mapatumumab in patients with relapsed/refractory non-Hodgkin’s lymphoma. Br. J. Cancer.

[103] Trarbach T., Moehler M., Heinemann V., Kohne C.H., Przyborek M., Schulz C., Sneller V., Gallant G., Kanzler S. (2010). Phase II trial of mapatumumab, a fully human agonistic monoclonal antibody that targets and activates the tumour necrosis factor apoptosis-inducing ligand receptor-1 (TRAIL-R1), in patients with refractory colorectal cancer. Br. J. Cancer.

[104] Bouralexis S., Findlay D.M., Evdokiou A. (2005). Death to the bad guys: Targeting cancer via Apo2L/TRAIL. Apoptosis.

[105] Plummer R., Attard G., Pacey S., Li L., Razak A., Perrett R., Barrett M., Judson I., Kaye S., Fox N.L. (2007). Phase 1 and pharmacokinetic study of lexatumumab in patients with advanced cancers. Clin. Cancer Res.

[106] Wakelee H.A., Patnaik A., Sikic B.I., Mita M., Fox N.L., Miceli R., Ullrich S.J., Fisher G.A., Tolcher A.W. (2010). Phase I and pharmacokinetic study of lexatumumab (HGS-ETR2) given every 2 weeks in patients with advanced solid tumors. Ann. Oncol.

[107] Campana D., Janossy G., Bofill M., Trejdosiewicz L.K., Ma D., Hoffbrand A.V., Mason D.Y., Lebacq A.M., Forster H.K. (1985). Human B cell development. I. Phenotypic differences of B lymphocytes in the bone marrow and peripheral lymphoid tissue. J. Immunol.

[108] Salvatore G., Beers R., Margulies I., Kreitman R.J., Pastan I. (2002). Improved cytotoxic activity toward cell lines and fresh leukemia cells of a mutant anti-CD22 immunotoxin obtained by antibody phage display. Clin. Cancer Res.

[109] Weldon J.E., Pastan I. (2011). A guide to taming a toxin—Recombinant immunotoxins constructed from Pseudomonas exotoxin A for the treatment of cancer. FEBS J.

[110] Alderson R.F., Toki B.E., Roberge M., Geng W., Basler J., Chin R., Liu A., Ueda R., Hodges D., Escandon E. (2006). Characterization of a CC49-based single-chain fragment-β-lactamase fusion protein for antibody-directed enzyme prodrug therapy (ADEPT). Bioconjug. Chem.

[111] Kreitman R.J., Tallman M.S., Robak T., Coutre S., Wilson W.H., Stetler-Stevenson M., Fitzgerald D.J., Lechleider R., Pastan I. (2012). Phase I trial of anti-CD22 recombinant immunotoxin moxetumomab pasudotox (CAT-8015 or HA22) in patients with hairy cell leukemia. J. Clin. Oncol.

